# A case report of recurrent thyroid inflammatory myofibroblastic tumor and its metastasis in soft tissue

**DOI:** 10.1097/MD.0000000000008485

**Published:** 2017-11-10

**Authors:** Jiajia Duan, Ying Wang

**Affiliations:** a Department of Interventional Radiology, China Meitan General Hospital; b Department of Pathology, Beijing Chaoyang Hospital of Capital Medical University, Beijing, China.

**Keywords:** inflammatory myofibroblastic tumor, metastasis, plasma cell granuloma, recurrence, thyroid

## Abstract

**Rationale:**

Inflammatory myofibroblastic tumor (IMT) is a neoplasm of low malignant potential. The most frequent site of IMT is in the lung, whereas recurrent and metastasis of thyroid IMT has been seldom reported.

**Patient concerns:**

A 57-year-old male presented with a 3-year history of painless thyroid mass. The physical examination revealed a diffusely enlarged thyroid which was firm. The thyroid function and antibodies were normal. Thyroid ultrasound revealed a hypoechoic mass in the left lobe and heterogeneous echo in the right lobe. Neck computed tomography showed a diffused enlargement of thyroid with the homogeneously low intensity and the moderate enhancement.

**Diagnoses:**

A diagnosis of thyroid IMT was made according the postoperative histological and immunohistochemical analysis.

**Interventions:**

The patient underwent subtotal thyroidectomy. Seventeen months after the surgery, the patients presented with a firm nodule of right adductor magnus and a relapsing mass of thyroid. Needle core biopsy of the thyroid mass suggested the relapsing of thyroid IMT. The mass excision of the right adductor magnus was performed and an IMT was confirmed by histopathology. The patient underwent thyroid radiation therapy and steroid therapy.

**Outcomes:**

The size of the tumor was smaller than the preradiation size and the patient is now under follow-up.

**Lessons:**

This is the seldom reported patient with recurrent thyroid IMT with metastasis. IMT of the thyroid is an unusual but distinct disease entity. The clinical and radiological features are not specific and its diagnosis is based on the histological features. Although tumor resection and radiation seem to be effective, no standard treatment for such disease has been established.

## 1 Introduction

Inflammatory myofibroblastic tumor (IMT), also known as inflammatory pseudotumour^[1]^ or plasma cell granuloma,^[2]^ is a rare disease reported to arise in various organs with uncertain therapy and prognosis. The World Health Organization (WHO) defined IMT as a soft tissue tumor.^[3]^ The most frequent site of IMT is in the lung or the upper respiratory tract, and the extrapulmonary IMT has also been reported at varied anatomic sites, mainly soft tissues and viscera.^[4,5]^ Head and neck lesions represent 14% to 18% of the extrapulmonary IMT cases.^[6]^ Its location in the thyroid is exceedingly rare. To our knowledge, the recurrent thyroid IMT or its metastasis has been seldom reported in the literature. This report presents a case of primary thyroid IMT with its recurrence and soft tissue metastasis.

## 2 Patient

Written informed consent was obtained from the patient for publication of this case report and accompanying images. In April 2011, a 57-year-old male presented to our hospital with a little hoarseness of 2-month duration and a 3-year history of painless thyroid mass. The thyroid mass was slowly progressive with no associated pain, dysphagia, dyspnea, shakiness, weight loss or increase, or emotional change or pressure symptoms. There was no specific past medical history and family history. He has a history of a-pack-of-cigarette-smoking (20 cigarettes per day) and a-500g-Chinese-liquor-drinking per day for 40 years. His general physical examination revealed a diffusely enlarged thyroid which was firm with the hazy margin and moved with deglutition. No enlarged cervical lymph nodes were detected on palpation. The rest of the physical examination was unremarkable.

Laboratory tests were as follows: white blood cell (WBC) count 6.19 × 10^9^/L (normal range: 4–10 × 10^9^/L) (64.2% neutrophils [normal range: 50%–70%], 22.9% lymphocytes [normal range: 20%–40%], 5.9% monocytes [normal range: 3%–8%], 6.4% eosinophils [normal range: 0.5%–5%], and 0.6% basophils [normal range: 0%–1%]), hemoglobin concentration 140 g/L (normal range: 120–160 g/L), platelet count 300 × 10^9^/L (100–300 × 10^9^/L), hematocrit 38.7% (normal range: 40%–50%). Thyroid function was normal (triiodothyronine 0.73 ng/mL [normal range: 0.7–2.0 ng/mL], thyroxine 6.61 μg/dL [normal range: 4.6–12 μg/dL], free T4 1.06 ng/dL [normal range: 0.7–1.9 ng/dL], free T3 2.13 pg/mL [normal range: 1.45–3.48 pg/mL], thyroid-stimulating hormone 3.395 μIU/mL [normal range: 0.27–4.2 μIU/mL]). Antithyroid peroxidase (0.19 IU/mL) (normal range: 0–60 IU/mL) and antithyroglobulin (0.64 IU/mL) (normal range: 0–115 IU/mL) were also within normal limits. The lactate dehydrogenase and serum β2-microglobulin were normal.

**Figure 1:**
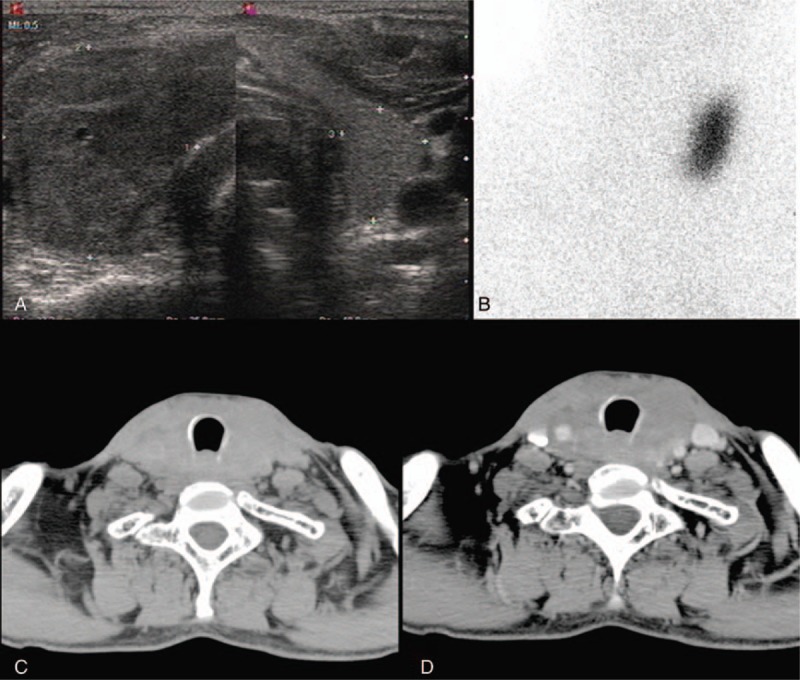
Clinical radiological data. (A) Thyroid ultrasound revealed a hypoechoic mass in the left lobe and heterogeneous echo in the right lobe. (B) 99TcmO-4 thyroid nuclear imaging showed a cold nodule in the right lobe of thyroid. (C) CT revealed a diffused enlargement of thyroid with the homogeneously low intensity in the noncontrasted phase. (D) The slightly homogeneous enhancement of thyroid in contrasted phase on CT. CT = computed tomography.

Thyroid ultrasound revealed a hypoechoic mass in the left lobe and heterogeneous echo with microcalcifications in the right lobe (Fig. 1A). 99TcmO-4 thyroid nuclear imaging showed a cold nodule in the right lobe of the thyroid gland (Fig. 1B). Neck computed tomography (CT) revealed a diffused enlargement of thyroid with homogeneously low intensity in the noncontrasted phase (Fig. 1C), and progressive, homogeneous, moderate enhancement of the thyroid (Fig. 1D) was found.

**Figure 2:**
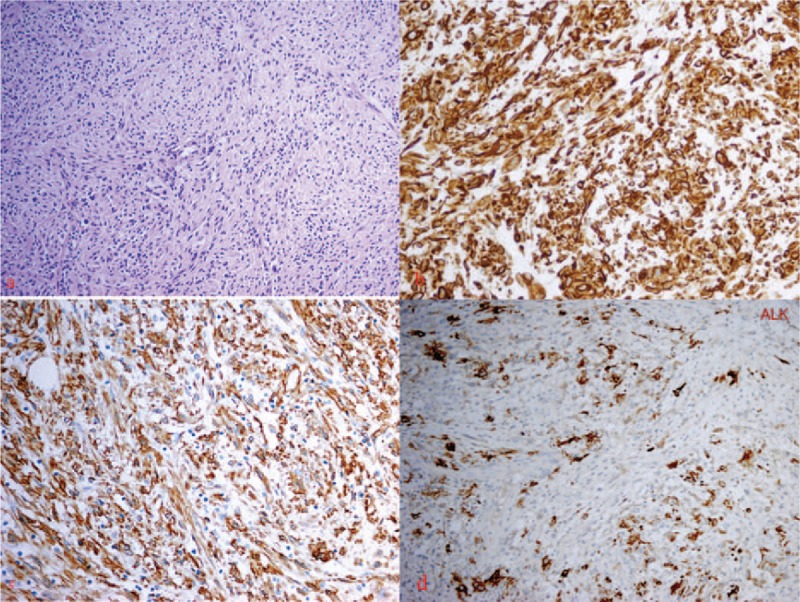
Histological study of thyroid mass. (A) Hematoxylin-eosin staining: the tumor is made up of a proliferation of spindle-shaped cells in a background of inflammatory cells (×200). (B) Immunohistochemical study: spindle-shaped cells positive for vimentin (×400). (C) Immunohistochemical study: spindle-shaped cells positive for smooth muscle actin (×400). (D) Immunohistochemical study: spindle-shaped cells positive for anaplastic lymphoma kinase (×400).

The patient underwent the subtotal thyroidectomy. During the operation, 1 firm mass in the right aspect measuring 4.0 cm × 3.2 cm × 1.5 cm and the other firm one in the left lobe measuring 3 cm × 2 cm × 2.5 cm were totally resected. Microscopic examination revealed a mixture of spindle cells and inflammatory cells including lymphocytes, plasma cells, immunoblasts, histiocytes, and fibrous tissue (Fig. 2A). Immunohistochemical staining demonstrated spindle cells positive for vimentin (Fig. 2B), smooth muscle actin (Fig. 2C), and anaplastic lymphoma kinase (ALK) (Fig. 2D). The tumor had a low Ki-67 proliferation. These features are consistent with an IMT. After surgery, the patient was discharged at the fifth postoperative day, and the patient refused any further treatment.

**Figure 3:**
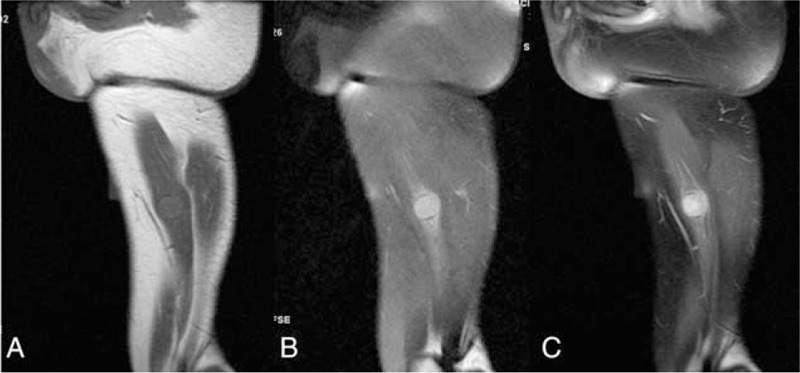
Magnetic resonance imaging (MRI) of the nodule in the right adductor magnus. (A) T1WI showed the homointensity of the nodule in the right adductor magnus. (B) T2WI showed the moderately high intensity. (C) Contrasted-MRI showed a homogeneous enhancement.

**Figure 4:**
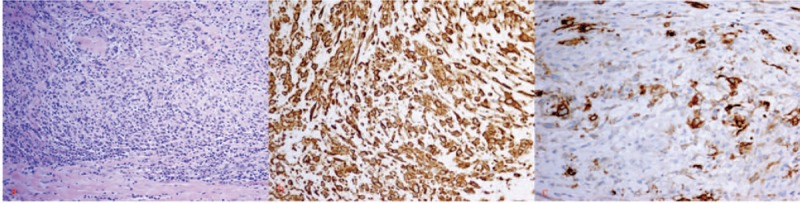
Histological study of right thigh mass. (A) Hematoxylin-eosin staining: the tumor is made up of a proliferation of spindle-shaped cells in a background of inflammatory cells (×100). (B) Immunohistochemical study: spindle-shaped cells positive for vimentin (×400). (C) Immunohistochemical study: spindle-shaped cells positive for anaplastic lymphoma kinase (×400).

On postoperative outpatient follow-up, the patient complained of a firm nodule in the right thigh and a relapsing mass of thyroid on September 2012. He confessed he did not stop heavy smoking and drinking after his discharge. Neck CT showed a mass of thyroid with a homogeneous low intensity. Magnetic resonance imaging demonstrated a 2.5 cm × 2.6 cm × 3.0 cm nodule in the right adductor magnus which demonstrated a moderate intensity on T1WI (Fig. 3A) and moderately high intensity on T2WI (Fig. 3B), and had a homogeneous enhancement (Fig. 3C). Subsequently, a needle core biopsy of the thyroid mass was taken and spindle cell proliferation was reported, which suggested the recurrence of thyroid IMT. A radical mass excision of the right thigh nodule was performed on February 2013 for the purpose of diagnosis and treatment, and an IMT was confirmed by histopathology, which suggested the metastasis of IMT (Fig. 4).

The patient quit smoking and drinking, and he underwent thyroid radiation therapy with a prescribed fraction dose of 2 Gy and total dose of 10 Gy, and oral steroid therapy. At present, the size of the thyroid tumor is smaller than the preradiation size and the patient is under follow-up.

## 3 Discussion

In this case, we reported the recurrence and distant metastasis of a thyroid IMT. IMT was originally first reported in the lungs.^[7]^ IMT used to be called inflammatory pseudotumour or plasma cell granuloma, so it was widely considered to be benign growths. Recent clinical observations and molecular data indicate that IMT is 1 kind of true neoplasm with low malignant potential. Its accurate diagnosis is based on the histopathologic and immunohistochemical analysis from a resected tumor.^[2,8]^ Hematoxylin-eosin staining of the resected specimen showed the proliferation of spindle-shaped cells in a background of inflammatory cells. Immunohistochemical study is helpful in diagnosing and distinguishing IMT from other types of tumors, which usually show positive staining for vimentin and smooth muscle actin, as in our patient.

According to WHO classification,^[3]^ IMTs are classified into 3 basic histological patterns: (1) a myxoid/vascular pattern; (2) a compact spindle cell pattern; (3) a hypocellular fibrous (fibromatosis-like) pattern. Thyroid involvement by IMT is a rare occurrence. Although 19 cases were reported to be the thyroid IMT (Table 1),^[9–26]^ only 2 reported cases^[23,24]^ exhibited the morphologic features of inflammatory myofibroblastic tumor in the compact spindle cell pattern. Our case presented as a painless swelling of the thyroid, which generally consisted of mostly uniform-appearing spindle cells accompanied by variable numbers of lymphocytes, neutrophils, plasma cells, and eosinophils. Immunohistochemical staining confirmed the myofbroblastic phenotype of the spindle cells, which are typically reactive to vimentin and smooth muscle actin, whereas reactivity to CD34 is negative. With this histopathological pattern, the thyroid IMT of our case was in a compact spindle cell pattern. The other reported cases^[9,10,12–22,25,26]^ had shown the prominent plasma cell infiltrate within a variable degree of fibrous stroma and were called plasma cell granuloma. They were quite different from our case. At present, we are not sure which histological pattern the thyroid plasma cell granuloma belongs to.

**Table 1:**
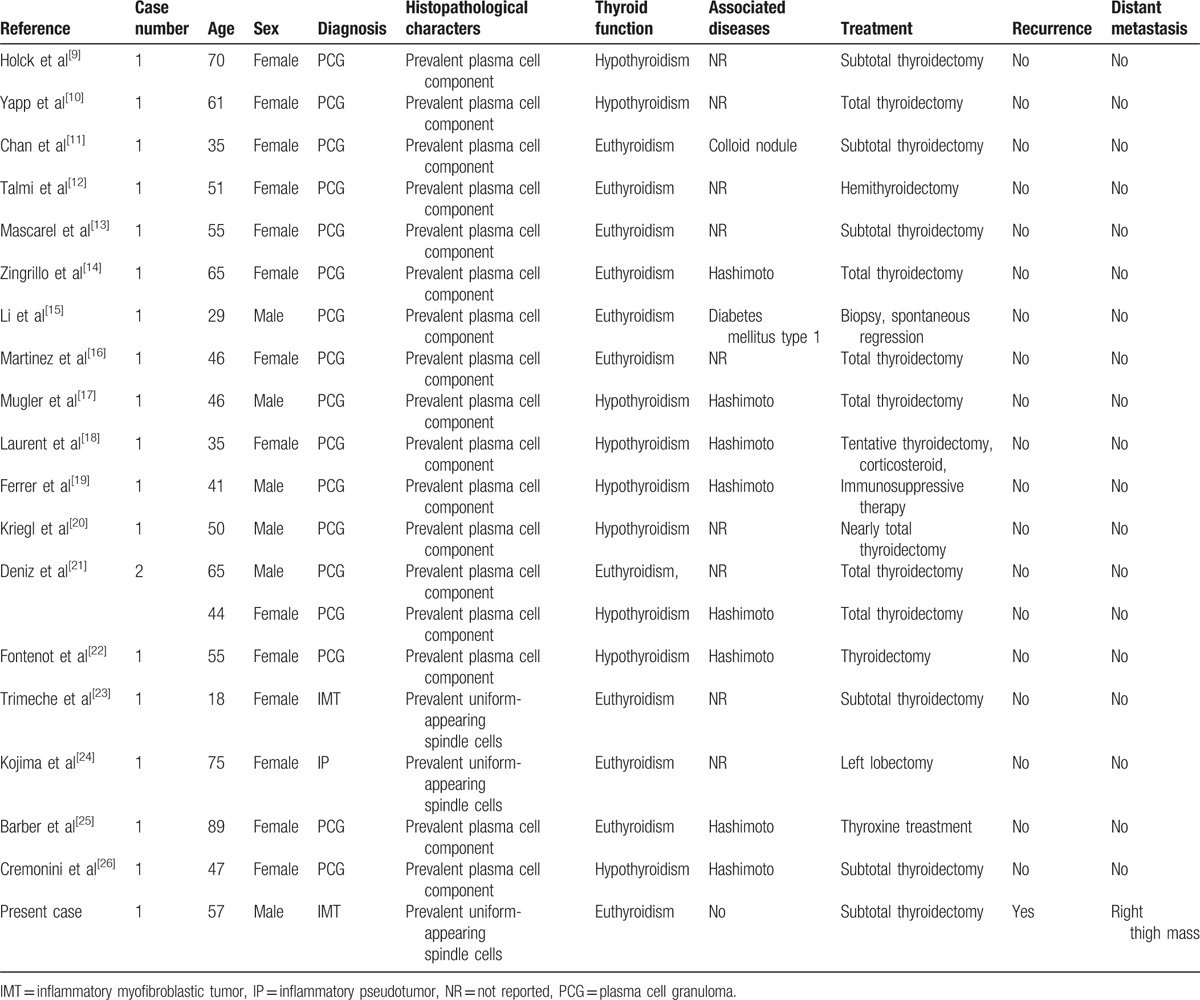
Clinical information of thyroid Inflammatory myofibroblastic tumor in the English literature.

The epidemiology of thyroid IMT remains unknown. Children and youth are mostly reported to be involved into other extropulmonary IMT.^[1,4]^ Among 20 patients with thyroid IMT,^[9–26]^ the age is 51.7 ± 16.6 years with a median age of 50.5 years. About 70% patients were women. Eight of the 17 thyroid plasma cell granulomas were associated with Hashimoto thyroiditis.^[14,17–19,21,22,25,26]^ Two reported cases with IMT had no Hashimoto thyroiditis.^[23,24]^ Also, there was no associated thyroid disorder in our present case. Another questionable point is whether heavy smoking or drinking is associated with IMT or not.

Inflammatory myofibroblastic tumor is a rare neoplasm that harbors an ALK gene rearrangement in the majority of cases. Immunohistochemical study demonstrated positivity for ALK in tumor cells from 50% of IMT cases.^[27–29]^ No ALK-positive cells were shown in those reported thyroid IMTs that included 17 plasma cell granulomas and 2 IMTs. On the contrary, our present thyroid IMT demonstrated the ALK-positive spindled cells.

Inflammatory myofibroblastic tumors are classified as tumors of intermediate risk, due to a small risk for local recurrence and distant metastasis. Time interval of recurrence ranged from several months to 9 years in extrothyroid tissue.^[29]^ At present, no recurrence and metastasis of the thyroid IMTs was reported. But our case manifested a recurrence and metastasis after subtotal thyroidectomy. This may be due to the fact that aggressive forms are reported to be ALK-1-positive.^[30]^

There are no specific signs or symptoms related to thyroid IMT. Most of them are painless mass with euthyroidism. In our case, thyroid CT showed a diffused enlargement of thyroid with the homogeneously low intensity in the noncontrasted phase, and progressive, homogeneous, moderately enhanced intensity of thyroid in the contrasted phase. We can easily differentiate IMT from thyroid carcinoma and infection thyroiditis in CT manifestation. But the primary thyroid lymphoma has similar CT manifestation. So, our primary diagnosis was the suspected thyroid lymphoma. The difference of CT manifestation between thyroid IMT and lymphoma still needs a further research.

Owing to the rarity of thyroid IMT, there has been no evidence, to date, regarding the optimal management. The majority of patients underwent either total/subtotal thyroidectomy or lobectomy. No anti-inflammatory therapy, chemotherapy, and radiation therapy has been tried, because no recurrence or metastasis was reported. In our case, the recurrent tumor became much smaller by radiation therapy and oral steroid therapy.

## 4 Conclusions

Inflammatory myofibroblastic tumor of the thyroid is a very rare but distinct disease entity. The old female seems mostly to be involved. The clinical and radiological features are not specific, and its diagnosis is based on the histological features. Although tumor resection and radiation seem to be effective in resistive disease, no standard treatment for such disease has been established.
